# Guidance for infectious disease care in the face of human conflict: a case-based narrative review

**DOI:** 10.1017/ash.2025.170

**Published:** 2025-05-13

**Authors:** Tessa Adžemović, Rachel Croxton, Payal Patel, Joseph Ladines-Lim, Elizabeth Scruggs-Wodkowski

**Affiliations:** 1 Department of Medicine, Brigham and Women’s Hospital, Boston, MA, USA; 2 Boston Children’s Hospital, Boston, MA, USA; 3 Department of Internal Medicine, Michigan Medicine, University of Michigan, Ann Arbor, MI, USA; 4 Infectious Diseases, Intermountain Health, Murray, UT, USA; 5 Division of Infectious Diseases, Department of Medicine, Penn Medicine, University of Pennsylvania, Philadelphia, PA, USA; 6 Infectious Diseases, Veterans Affairs Ann Arbor Healthcare System, Ann Arbor, MI, USA; 7 Division of Infectious Diseases, Department of Internal Medicine, Michigan Medicine, University of Michigan, Ann Arbor, MI, USA

## Introduction

Armed conflict has been linked to the spread of antimicrobial resistance (AMR),^1^ including recent reports in Syria,^2,3^ Ukraine,^4^ and Gaza.^5^ Contributing factors that arise from conflict include but are not limited to overcrowding, limited healthcare access, poor sanitation and hygiene, inadequate infection control measures, and antimicrobial dispensations without regulation or oversight.^1^ Conflict results in internal displacement of persons, which has been associated with increased carriage of multidrug-resistant organisms (MDROs).^6^ The degradation of healthcare infrastructure and disruption of vaccine programs as a result of armed conflict has also resulted in increased incidence of vaccine-preventable illnesses, such as poliomyelitis and measles, and other infectious disease outbreaks, such as tuberculosis and cholera.^7^

Clinicians who now care for these populations in the United States (US) face numerous challenges: limited experience for certain diseases, scant AMR surveillance data in other countries to inform risk stratification for MDRO carriage, and difficulties inherent in treating refugee and migrant populations due to patients’ struggles with acclimation and traumatic adverse experiences, among many other reasons.^8,9^ Recent literature has addressed policy implications for delivering care in these settings directly^10–12^ and there have been some efforts to establish guidelines for clinical care,^13^ including guidance from the Centers for Disease Control and Prevention (CDC) for performing mandatory health screenings and educational materials on topics such as COVID-19.^14^ However, there still remains minimal guidance on caring for these patients in the community, particularly from an Infectious Diseases perspective.

In this narrative review, we describe the state of emerging infections in conflict zones and the downstream sequelae of increased human displacement and migration through three characteristic fictionalized cases. We aim for these cases to be instructive in providing practical guidance on caring for this vulnerable community.

## Case #1: Multidrug-resistant bacterial infection

A 52-year-old Ukrainian woman comes to the Infectious Diseases clinic. She has been referred by her primary care provider at the nearby federally qualified health center. She arrived in the (US) five weeks ago. She is otherwise healthy but shares with you that she was hospitalized six months ago in Ukraine after she sustained a blast injury from shrapnel, resulting in a wound to her right lower extremity that has failed to heal completely. Examination of her right ankle reveals a chronic erosive ulcer with drainage and mild surrounding erythema. The patient received antibiotics in Ukraine but is not certain what kind and is no longer on treatment.

### How do you proceed?

Refugees and migrants arriving from Ukraine require careful review of all vaccine records, medical notes (if available), and a full history and physical exam. Use of a certified medical translator is paramount. General vaccination requirements for Ukrainian refugees include measles, polio, and COVID-19, but also pertussis, diphtheria, tetanus, and hepatitis A, amongst others.^15,16^ All patients should undergo tuberculosis screening with an interferon gamma receptor antigen (IGRA) blood test and be referred to the local health department if they exhibit signs or symptoms of active tuberculosis (i.e., cough, fever, weight loss, failure to thrive, night sweats, hemoptysis).^15,16^ Patients should also be screened at intake for hepatitis B, hepatitis C, and HIV.

While not a medical requirement for resettlement in the US, clinicians should strongly consider screening patients from countries with increasing rates of multidrug resistance (MDR), including human conflict zones such as Ukraine. Among others, increasing numbers of MDR *Klebsiella pneumoniae* and *Acinetobacter baumanii*, both carbapenem resistant, have been detected in patients both within Ukraine and resettled elsewhere. Risk factors include war-related injuries, prior hospitalization, and prior antibiotic use.^17,18^ The European Centre for Disease Prevention and Control (ECDC) recommends pre-emptive isolation and MDRO screening, in particular carbapenem-resistant *Enterobacterales* (CRE), for patients with traumatic wounds.^19^ In this scenario, if screening reveals infection with an MDRO such as CRE, a multidisciplinary treatment approach is recommended. This includes consultation with an Infectious Diseases specialist for selection and monitoring of appropriate antimicrobial therapy, such as meropenem-vaborbactam, ceftazidime-avibactam, or imipenem-cilastatin-relebactam.^20^ Wound care, debridement, and need for reconstruction should also be coordinated with a surgical subspecialty, e.g., Plastic Surgery.

## Case #2: Tuberculosis in a person living with HIV (PLHIV)

A 29-year-old Congolese man presents to the asylum clinic for a medical affidavit. He has been residing in a shelter. He has a history of HIV diagnosed in a Ugandan refugee camp and comments that he has had a cough for the last couple of months. He is not currently receiving antiretroviral therapy and identifies as a man who has sex with men (MSM). He describes being forcibly detained in the Democratic Republic of the Congo (DRC) and sustaining burns and whippings at that time. Physical exam reveals a cachectic-appearing male, palpable cervical lymphadenopathy, bilateral crackles upon bilateral auscultation, multiple linear hypertrophic scars over the lumbar spine, and several healing puncture wounds on the bilateral buttocks.

### How do you proceed?

People living with HIV (PLHIV) are at risk of various opportunistic infections including *Mycobacterium tuberculosis*, *Cryptococcus neoformans*, and *Pneumocystis jirovecii* pneumonia, among others. The patient in this case raises suspicion for tuberculosis (TB). In 2021, it was estimated that there were 10.6 million people who developed TB, 6% of whom were PLHIV.^21^ Notably, 30 high TB burden countries account for 86% of all TB in the world, including the DRC, Uganda, South Africa.^22^ Concerningly, human conflict is associated with decreased notification rates of TB and up to 20 times increase in TB.^23^

Because of the immune dysregulation in HIV, screening for TB in PLHIV is crucial. A four-symptom screening tool (current cough, fever, weight loss, or night sweats) should be used.^24^ If any one of these four symptoms is positive, then further testing should be performed, including chest radiography and sputum samples for acid-fast bacillus (AFB) smear, culture, and nucleic acid amplification testing (NAAT). Because of the high incidence of latent TB in PLHIV, the CDC requires AFB smear and culture to be performed even in asymptomatic patients in the refugee population.^25^

Treatment typically consists of two months of an intensive phase with isoniazid, rifampin, pyrazinamide, and ethambutol given daily, with four months of continuation phase (isoniazid, rifampin, and ethambutol) given daily.^26,27^ Additionally, the rates of multidrug-resistant TB (MDR-TB), which is resistant to both isoniazid and rifampin with or without resistance to other first-line anti-TB agents, are increasing.^28^ PLHIV are at high risk of MDR-TB due to delays in diagnosis, higher mycobacterial loads, malabsorption of isoniazid, rifampin, and pyrazinamide, and nosocomial transmission of MDR-TB.^29^ Notably, human conflict disrupts healthcare services, and consequently TB treatment, leading to an increase in MDR-TB. NAAT allows for rapid identification of MDR-TB while cultures are in process. All TB therapy should be coordinated with local health department guidance, and MDR-TB is best managed by an Infectious Diseases specialist to assist with regimen selection. Notably, there is increased risk of TB conversion in migrant populations within the first five years of arrival to a low-TB-incidence country from a high-TB-incidence country.^30^

The incidence of TB in people experiencing homelessness is ten times greater than that of the general population.^31^ This patient resides in a shelter and will need to be isolated and rapidly treated in order to prevent transmission to others. In collaboration with local public health officials, residents of the shelters should undergo contact investigations and screenings due to their higher likelihood of exposure to TB. Additionally, Directly Observed Therapy is highly recommended to ensure adherence to the treatment regimen.

Beyond understanding the medical issues affecting asylum seekers, it is important for providers to be aware of the unique psychosocial challenges faced by this population. Our previous qualitative work involved interviews with providers working at clinics in Southeast Michigan that care for large populations of patients from asylee, refugee, and immigrant backgrounds.^8,9^ One emergent theme from these interviews was the nuance and subtleties with which psychosocial trauma may manifest. Our work revealed some patients exhibited increased levels of stress, post-traumatic stress disorder, and homesickness. Trauma may present as nonspecific physical symptoms, such as abdominal pain, headaches, or fatigue, without a clear organic cause. All asylum seekers should be screened for evidence of torture, human trafficking, and sexual and gender-based violence. Comprehensive care for patients who arrive in the US from other countries ought to be trauma-informed and recognize the psychological impact of displacement, for which various other resources can provide guidance.^32^

## Case #3 Poliomyelitis

A 24-month-old from Gaza presents for follow-up care in your clinic. He was diagnosed with poliomyelitis at the age of 10 months old and has lower extremity paraplegia. On exam, he has bilateral muscle atrophy, diffuse hyporeflexia, and an ataxic gait.

### How do you proceed?

Poliomyelitis, a vaccine-preventable illness, has resurged in conflict zones. While polio was previously eradicated from Palestine, the World Health Organization detected poliovirus (vaccine-derived poliovirus type 2) in wastewater in June 2024 due to destruction of healthcare infrastructure during the current humanitarian crisis.^33,34^ In August 2024, the first case was confirmed in a ten-month-old unvaccinated child. Factors such as the dissolution of healthcare services and routine immunization programs contributed to the re-emergence of polio. Afghanistan and Pakistan remain polio-endemic countries.

The virus has three serotypes and spreads via the oral-fecal route. Though carriers may be asymptomatic, 1 in 200 to 1 in 2,000 will develop paralysis. Severe cases can result in quadriplegia, respiratory failure, and rarely death.^35^

The inactivated polio vaccine was developed by Jonas Salk, who licensed the first version in 1955, with wide distribution in the US. The oral poliomyelitis vaccine was designed by Sabin and contains live attenuated viral strains. Most recently, the novel oral polio vaccine type 2 has been used in an oral mass vaccination campaign in Gaza, with successful administration rounds in September and October 2024 and without any additional cases reported since August 2024.^36^ Still, ongoing transmission has been confirmed as of January 2025 via environmental sampling,^36^ underscoring the difficulty of protecting vulnerable populations in the face of massive conflict and dismantled public health infrastructure.^33,37^

For this patient in question, it is important to understand the historical legacy of his illness and ensure that he and his family receive appropriate ancillary care, which may include referrals to physical therapy, orthotics, and adaptive equipment. He and his siblings should receive age-appropriate vaccines, including the polio vaccine, if indicated.

## Conclusion

Clinicians caring for patients who have been affected by human conflict face myriad infectious disease challenges, including increased risk of MDRO carriage and subsequent infection, vaccine-preventable diseases, and other infections not endemic to the US such as TB. Furthermore, many patients affected by human conflict experience displacement and sometimes persecution; subsequently, they often experience trauma and psychosocial distress, which may have diverse clinical manifestations without a clear organic cause.

**Figure 1.**
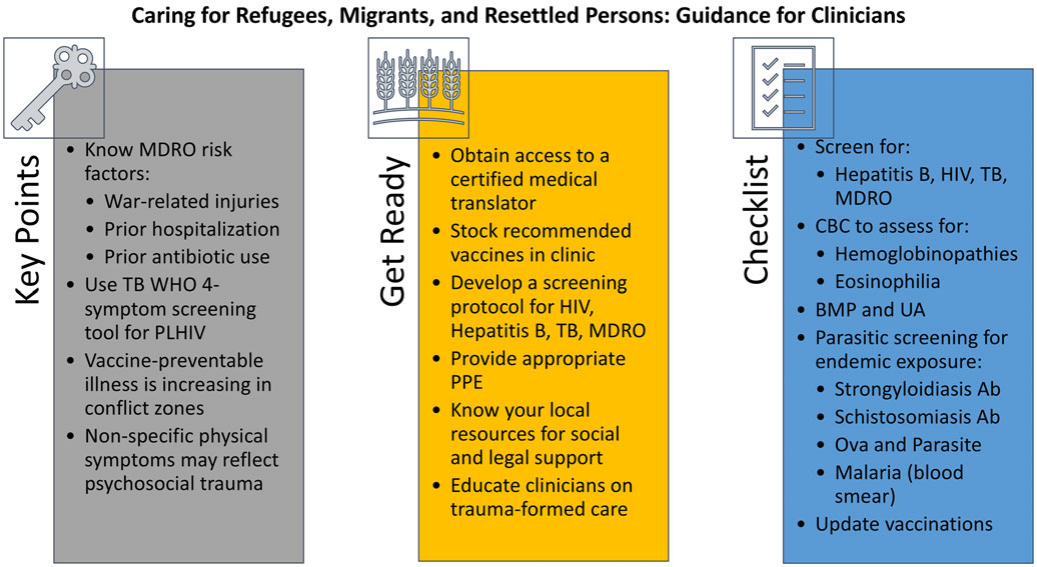
Takeaway points from cases and other infectious disease care guidance. BMP: basic metabolic panel; CBC: complete blood count; MDRO: multidrug-resistant organism; PLHIV: people living with HIV; PPE: personal protective equipment; TB: tuberculosis; UA: urinalysis; WHO: World Health Organization.

In our three cases of likely multidrug-resistant wound infection, TB/HIV, and morbid sequelae of poliomyelitis infection, we aimed to equip clinicians with knowledge and tools to best care for these patients. We have listed recommendations and learning points for each case in Figure 1. In general, clinicians should prioritize vaccinations (priority for measles/mumps/rubella, polio, and COVID-19, but with considerations for any and all others that may be indicated); perform infectious screenings (e.g. IGRA, MDROs); coordinate with the local health department when needed (e.g. TB care); involve social and legal services to address other determinants of health; and use a trauma-informed approach when addressing symptoms that may have resulted from adverse experiences common in this population.

Particularly with the new administration and as geopolitical events continue to unfold, we hope that this serves as a practical reference guide for clinicians caring for populations beset by human conflict.
